# Comprehensive analysis of treatment response phenotypes in rheumatoid arthritis for pharmacogenetic studies

**DOI:** 10.1186/s13075-017-1299-8

**Published:** 2017-05-12

**Authors:** Kristopher A. Standish, C. Chris Huang, Mark E. Curran, Nicholas J. Schork

**Affiliations:** 1 Biomedical Sciences Graduate Program, University of California, San Diego, Gilman Drive, La Jolla, 92092 CA USA; 2 Human Biology, J. Craig Venter Institute, 4120 Capricorn Lane, La Jolla, 92092 CA USA; 3 Network Pharmacology and Biomarkers, Janssen R&D LLC, Springhouse, PA USA

**Keywords:** Rheumatoid arthritis, Pharmacogenetics, Genetics, Heritability, Phenotype, Placebo

## Abstract

**Background:**

An individual patient’s response to a particular drug is influenced by multiple factors, which may include genetic predisposition. Pharmacogenetic studies attempt to discover and estimate the contributions of genetic variants to the variability in response to a drug treatment. The task of identifying the genetic contribution is often complicated by response phenotypes that are based on imprecise or subjective clinical observations. Because the success of a pharmacogenetic study depends on the analysis of a heritable phenotype, it is important to identify phenotypes with a significant heritable component to ensure reliable and reproducible results in subsequent genetic association studies.

**Methods:**

We retrospectively analyzed data collected from 436 rheumatoid arthritis patients treated with golimumab during the phase III GO-FURTHER study. We investigated the reliability of several potential response outcomes after golimumab treatment. Using whole-genome sequencing of the clinical trial cohort, we estimated the heritability of each potential outcome measure. We further performed a longitudinal analysis of the clinical data to estimate variability of outcome measures over time and the degree to which each response metric could be confounded by placebo response.

**Results:**

We determined that the high degree of within-patient variation over time makes a single follow-up visit insufficient to assess an individual patient’s response to golimumab treatment. We found that different potential response outcomes had varying degrees of heritability and that averaging across multiple follow-up visits yielded higher heritability estimates than single follow-up estimates. Importantly, we found that the change in swollen and tender joint counts were the most heritable outcome metrics we tested; however, we showed that they are also more likely to be confounded by a placebo response than objective phenotypes like the change in C-reactive protein levels.

**Conclusions:**

Our rigorous approach to finding robust and heritable response phenotypes could be beneficial to all pharmacogenetic studies and may lead to more reliable and reproducible results.

**Trial Registration:**

Clinicaltrials.gov NCT00973479. Registered 4 September 2009.

**Electronic supplementary material:**

The online version of this article (doi:10.1186/s13075-017-1299-8) contains supplementary material, which is available to authorized users.

## Background

Rheumatoid arthritis (RA) is a complex, chronic, and debilitating autoimmune disorder characterized by stiff and painful joints, chronic inflammation, synovitis, irreparable joint damage, and the presence of auto-antibodies. Although its precise etiology is unclear, RA has been shown to have a strong genetic component, as concordance in monozygotic twins is 15–30% while the population prevalence is around 1%. Some have estimated the heritability for the disease to be as high as 50% [1, 2]. A majority of the genetic susceptibility can be attributed to polymorphisms at five amino acid residues in HLA-DRB1, -B, and -DPB1 [3]. In addition, close to 100 loci from non-HLA genes have been shown to contribute to disease susceptibility [4]. However, because the genetic variants cumulatively explain only about 18% of disease variance, a large environmental influence has yet to be clearly defined [5].

Despite an incomplete understanding of its etiology, a set of clinical features as well as laboratory measurements have allowed standardized diagnoses and assessment of treatment efficacy in RA [6, 7]. One commonly used metric is Disease Activity Score (DAS28 or DAS), which incorporates swollen and tender joints counts (SJC and TJC, respectively) out of 28 affected joints, the erythrocyte sedimentation rate (ESR) and a visual analog scale score for general health (VASGH) into a formula where a higher score (up to 10) indicates a more severe disease state [8, 9]. Variations of this score include the use of C-reactive protein (CRP) as an acute inflammation marker to replace ESR [10, 11]. Alternatively, categorical definitions of RA treatment response have been developed by the American College of Rheumatology (ACR). For example, ACR20 and ACR50 scores represent a 20% or 50% improvement of disease state post-intervention based on a combination of SJC, TJC, patient and physician global assessments, pain, disability, and an acute-phase reactant such as CRP [12, 13]. Similarly, the European League Against Rheumatism has developed guidelines for classifying patients’ treatment responses into good, moderate, and non-response based on the change in DAS from baseline [14]. The numerical DAS score, various forms of DAS-based categorical variables, and ACRs are commonly used as response metrics in RA clinical trials. Despite this, other measurements such as the Health Assessment Questionnaire and radiographic assessment of the affected joints may provide further evidence. Generally, each of these measurements has its own strength and limitations and several of them are often assessed in combination to gain a comprehensive view of the disease state or treatment effect.

The goal of a pharmacogenetic study is to establish the association between certain genetic variants and patients’ response to a therapy, and ultimately to estimate the contributions of the genetic variants to the variability of treatment response. While recent advances have made genotyping widely available and highly precise, phenotypic measurements are still limited by issues with precision and completeness, which is the ability of a measurement to fully represent the symptoms and underlying mechanisms of a complex disease such as RA. Consequently, pharmacogenetic studies can be confounded by imprecise, subjective, or narrow response phenotypes. For example, a class of anti-TNF *α* antibodies that includes etanercept, infliximab, golimumab, adalimumab, and certolizumab pegol are often used in conjunction with disease-modifying anti-rheumatic drugs (DMARDs), such as methotrexate (MTX), in RA patients who have an insufficient response to DMARDs alone. Evidence has been published supporting a role for variants in the genes *TNFA*, *TNFR1A*, *MED15*, *PTPRC*, *FcGR2A*, *FcGR3A* and others in influencing response to anti-TNF *α* treatment, but few associations have been successfully replicated [15–19]. The lack of replication may be due, in part, to the use of response phenotypes that exhibit insufficient heritability, i.e., the variance of these phenotypes cannot be explained by genetic variation, but rather by some other factors. The heritability of a phenotype can be estimated using sophisticated statistical methods such as those implemented in the Genome-wide Complex Trait Analysis toolkit (GCTA) [20].

Only two studies have estimated the heritability of DAS28, SJC, TJC, and ESR as outcome metrics to various anti-TNF *α* therapies [21, 22]. For example, an early study looked at a group of 762 RA patients treated specifically with anti-TNF *α* monoclonal antibodies. It suggested that SJC was the most heritable outcome metric (0.60), followed by ESR (0.53) and TJC (0.35), while the global health assessment score was the least heritable (0.14) [21]. Most recently, Umiceviv Mirkov, et al. used two methods to estimate the heritability of response to anti-TNF *α* agents in a cohort of 878 patients through 14 weeks of treatment. This study suggested that SJC (0.87) and TJC (0.82) had the highest heritability estimate while ESR (0.33) and VASGH (0.38) had the lowest estimates [22]. A close examination of the statistical methods used in these studies revealed several issues that may account for some of the apparent discrepancies. First, some of the phenotypes used to quantify patients’ drug responses exhibited skewed distributions that violate statistical assumptions of parametric hypothesis tests, resulting in unreliable *p* values. Second, most outcomes were determined at a single follow-up visit after treatment. Because of their imprecise and subjective nature, these response metrics vary over time, resulting in potentially unreliable estimates of drug response for any given patient. Finally, none of the published studies account for placebo response when interpreting their results. This oversight could result in an improvement in a patient’s disease state being incorrectly attributed to a treatment rather than some other unknown factor.

Here, we sought to identify robust, heritable phenotypes associated with anti-TNF *α* drug response using a set of clinical and genetic data collected from the GO-FURTHER study [23, 24]. We investigated several possible response metrics, including changes in DAS, SJC, TJC, and CRP levels after treatment with golimumab. We showed that outcomes determined from a single follow-up visit may not be sufficient to describe a patient’s true response to a treatment and demonstrated that using different outcomes to quantify a patient’s response would likely yield different results in subsequent association studies. We then estimated the heritability of each potential response measure and showed that outcomes averaged across several follow-up visits resulted in higher heritability estimates than those collected from a single follow-up visit. Finally, we estimated the degree to which a patient’s improvement in disease state could be attributable to placebo response rather than active treatment and showed that the relative magnitude of placebo response depends on which metric is used to estimate a patient’s disease state.

## Methods

### Clinical trial design and data collection

**Fig. 1:**
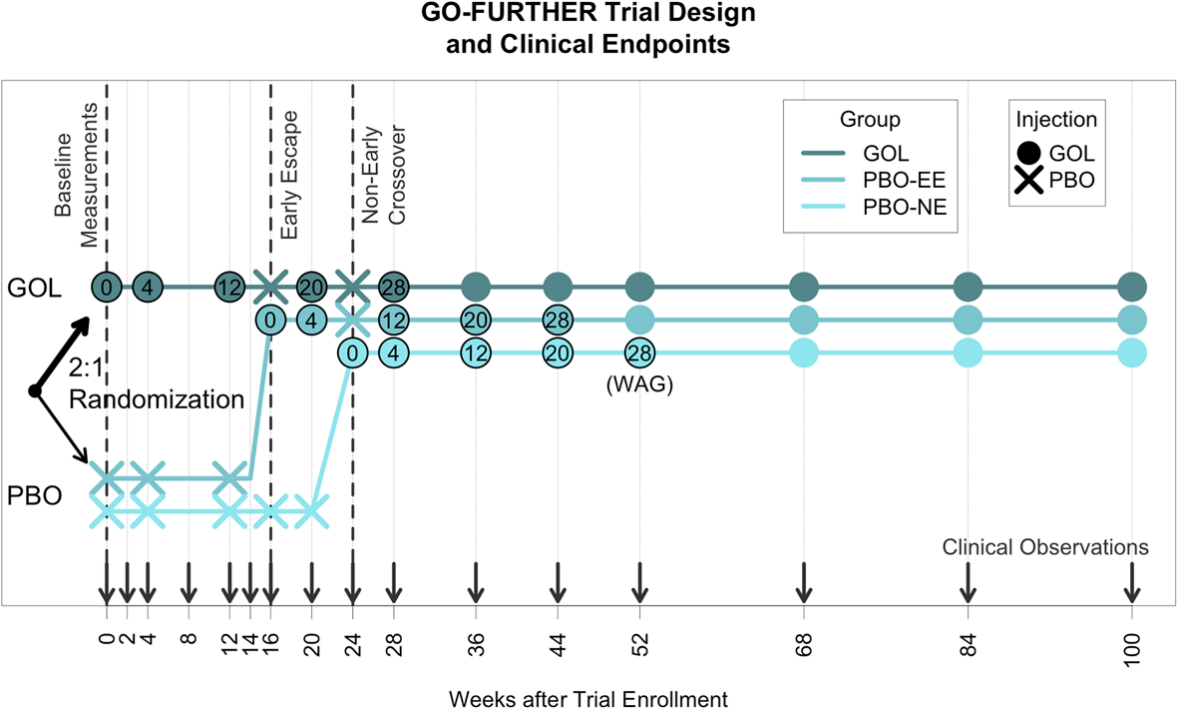
GO-FURTHER trial design and clinical endpoints. Longitudinal design for the GO-FURTHER phase III clinical trial. Patients were initially randomized to the GOL or PBO arms of the trial (2:1). GOL patients were initiated onto golimumab treatment at week 0. PBO patients were treated with a placebo at week 0 and were treated with golimumab at week 24 (PBO-NE) or week 16 (PBO-EE). Injections and clinical observations occurred as indicated. *EE* early escape, *GOL* golimumab, *NE* non-early escape, *PBO* placebo, *WAG* weeks after initial GOL treatment

We leveraged clinical and genetic data obtained from the GO-FURTHER phase III clinical trial for 436 patients with moderate to severe RA [23–25]. In brief, each patient was randomized to either treatment golimumab (GOL) or placebo (PBO) arms (2:1) at week 0 and followed for 100 weeks (Table 1, Fig. 1). Patients in the GOL arm were given 2 mg/kg of GOL intravenously at weeks 0 and 4 and every eight weeks thereafter. Patients in the PBO arm received PBO injections at weeks 0, 4, and 12. If a patient qualified for the early escape protocol (<10% improvement from baseline; PBO-EE), they were given intravenous GOL at weeks 16, 20, and every 8 weeks thereafter. If they did not qualify for early escape (PBO-NE), they were given PBO injections at weeks 16 and 20 and were treated with GOL at weeks 24 and 28 and every 8 weeks thereafter. All patients were on a MTX regimen prior to and throughout the study, despite any inadequate response to MTX as a mono-therapy previously. All patients, minus 15 early dropouts, were included in statistical analyses without the use of the last observation carried forward method (Table 1). Disease state was monitored at 16 predetermined time points over 100 weeks, during which CRP, SJC, and TJC were assessed and DAS was calculated (Fig. 1). By virtue of the trial design, there were four matched time points for all three groups after initial GOL treatment: 4 weeks, 12 weeks, 20 weeks, and 28 weeks after GOL treatment (WAG).

**Table 1:** Summary of clinical trial patients and arms

	GOL	PBO-NE	PBO-EE
Number of patients	287	99	50
Female (%)	231 (0.8)	72 (0.73)	41 (0.82)
Age (SD)	51.92 (11.94)	52.77 (11.17)	49.24 (11.84)
Disease duration (SD)	7.19 (6.82)	7.64 (8.16)	6.56 (6.06)
BMI (SD)	27.19 (5.74)	26.88 (5.36)	26.73 (6.65)
Initial DAS (SD)	5.97 (0.81)	5.87 (1.02)	5.93 (0.8)
RF positive (%)	265 (0.92)	91 (0.92)	45 (0.9)
ACPA positive (%)	262 (0.91)	92 (0.93)	48 (0.96)
Number of visits (SD)	15.03 (2.7)	14.65 (3.18)	15.76 (0.72)
Number removed (%) ^a^	5 (2)	10 (10)	0 (0)

GOL includes patients randomized to GOL treatment at week 0. PBO-NE includes patients randomized to PBO at week 0 who did not qualify for the early escape protocol. PBO-EE includes patients randomized to PBO at week 0 who qualified for the early escape protocol

*ACPA* anti-citrulinated peptide antibody, *BMI* body mass index, *DAS* Disease Activity Score, *EE* early escape, *GOL* golimumab, *NE* non-early escape, *PBO* placebo, *RF* rheumatoid factor, *SD* standard deviation

^a^Patients from any arm who dropped out within 4 weeks of initial GOL treatment were removed from analyses

The study protocol was approved by local institutional review boards and only trial patients who gave informed consent for the genetic study were sequenced. Whole blood was taken from patients according to the trial protocol and whole-genome sequencing and variant calling were performed as described in Standish, et al. [25]. Additional details about the clinical trial can be found at www.clinicaltrials.gov (NCT00973479) and in previous publications [23, 24].

### Statistical analysis

#### Individual response outcomes

We used the R suite for all statistical analyses involving clinical response data [26]. Summary response metrics from single follow-up clinical visits were calculated for each patient as the difference in disease state (e.g., DAS, CRP, SJC, and TJC) between two clinical visits. Response measures specified as 4, 12, 20, and 28 WAG were calculated as the difference between a patient’s disease state immediately prior to initial GOL injection and their disease state 4, 12, 20, or 28 weeks later, respectively (Fig. 1). Response metrics specified as FL represent the difference between a patient’s disease state during their first clinical visit (week 0) and their last clinical visit (week 100 or earlier if they dropped out prior to trial completion). Baseline disease state was used as a covariate for subsequent analyses of treatment response. For WAG response measures, measurements from the clinical visit immediately prior to initial GOL treatment were used as the baseline disease state. For FL response measures, the disease state at week 0 was used as the baseline.

#### Average response outcomes

Average response phenotypes were calculated from the repeated clinical measurements obtained on the individuals throughout the trial. Weighted mean disease state was calculated before and after treatment with GOL as the area under the response curve over time divided by the number of weeks. For patients in the GOL arm of the study, the single baseline measurement was taken as the pre-treatment disease state. Mean treatment response (MNa) was calculated as the difference between average disease states before and after GOL initiation. We further derived additional statistics that represented the average response of a patient, the patient-specific variance in response, and the patient disease trajectory over time, but these derived phenotypes did not contribute significantly to our understanding of individual patient response and were not considered further.

#### Parametric testing

To test these continuous response outcomes for normality, we used the Shapiro–Wilk normality test on the raw data, transformed data, or residuals after accounting for clinical covariates. We used the Breusch–Pagan test to assess the homoscedasticity of response variables against clinical covariates. When we regressed the change in disease state against the baseline disease state, a common covariate included in genetic association studies, we found that the residuals of the CRP, SJC, and TJC metrics violated the assumption of normality. After transforming the metrics in the same way, they are transformed to calculate DAS, which is the square root for SJC and TJC (rSJC and rTJC, respectively) or the logarithmic scale for CRP (lCRP). These data conformed better to the assumption of normality. In addition, we found that the assumption of homoscedasticity was violated when regressing the change in SJC and TJC against their respective initial values. As a result, permutations were used to determine reliable *p* values from subsequent heritability analyses.

#### Correlation between outcomes

To determine how correlated these potential response metrics were with one another, we calculated the pairwise correlation between response outcomes obtained from a single follow-up visit (e.g., WAG4_DAS vs WAG28_DAS) and averaged over multiple visits (e.g., WAG4_DAS vs MNa_DAS). We calculated the correlation amongst different metrics and follow-up visits after transforming the data to follow a normal distribution, so the Pearson correlation coefficient was used. Pairwise correlations are represented in a heat map where all negatively correlated values appear black.

#### Linear mixed model

We fit a linear mixed model to the transformed disease metrics (DAS, lCRP, rSJC, and rTJC) collected on patients throughout the clinical trial, including random effects to accommodate repeated measurements for each individual. Baseline disease state (intercept), GOL, and PBO were estimated as fixed effects to obtain population-level effect sizes (*β*_intercept_, *β*_GOL_, and *β*_PBO_, respectively). Random intercepts and GOL effects were used to obtain estimates for individuals in the cohort. For each clinical visit (approximately 16 visits per patient × 421 patients), the treatment protocol was defined for GOL (i.e., if they were on golimumab during a given observation) and PBO (i.e., if they were on placebo during a given observation) as binary dummy variables.

Within-patient variation over time was quantified from the residuals of the linear mixed model. For each patient, the root-mean-square error (RMSE) was calculated as a measure of variation that could not be accounted for by treatment with the active drug or PBO. Using the estimated fixed effects from the mixed model, we quantified the magnitude of the population-level PBO effect for each disease metric. We compared the respective PBO effect size estimates relative to baseline disease state and relative to the GOL effect size. The percentage improvement in disease state due to PBO response was calculated as 100×*β*_PBO_/ *β*_intercept_. The percentage of the GOL effect that can be attributed to PBO was calculated as 100×*β*_PBO_/ *β*_GOL_.

#### Heritability estimates and permutations

Heritability of the various response phenotypes described above was estimated using the GCTA software suite (v1.24.4) [20]. Single-nucleotide polymorphisms with Minor Allele Frequency (MAF) of <1% in our cohort were removed when calculating the genetic relationship matrix (GRM) for GCTA analysis. Initial disease state was used as a covariate when estimating the heritability of treatment response and the analysis was run with and without four principal components included as covariates. Because not all the linear regression assumptions were explicitly met, permutation tests were used to assess the statistical significance of the heritability estimates by randomly shuffling patients’ response phenotypes 1000 times and then re-running GCTA using the previously calculated GRM. *p* values were calculated by comparing the distribution of permuted likelihood-ratio test statistics with the likelihood-ratio test statistic from the actual data.

## Results

### Variability of potential response outcomes

**Fig. 2:**
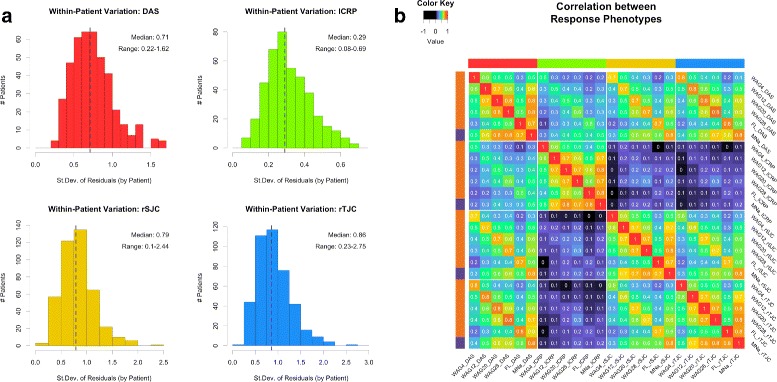
Variability in potential response outcomes. **a** Distribution of within-patient error from the mixed model using different outcome metrics. **b** Pairwise Pearson correlation between potential outcome measurements using different metrics and follow-up visits. *DAS* Disease Activity Score, *FL* difference between first and last measurements, *lCRP* log transform of C-reactive protein metric, *rSJC* square root of swollen joint count, *rTJC* square root of tender joint count, *St.Dev.* standard deviation

Technical error and biological factors can contribute to variation in a patient’s observed disease state. When trying to determine each patient’s response to GOL treatment, we found that a patient’s disease state varied from week to week. To better understand the variation present for each patient, we quantified within-patient variation for each patient that could not be explained by GOL or PBO treatment. After fitting a linear mixed model to outcome data collected from clinical visits throughout the trial, we calculated the RMSE from the residuals of each patient’s observations and found that many patients had clinically meaningful variation in disease state over the course of the trial. We found similar distributions of within-patient error across the population, regardless of which outcome metric was used (i.e., DAS, lCRP, rSJC, or rTJC; Fig. 2 a). The magnitude of unexplained variation in a patient, for DAS, ranged from 0.22 to 1.62 with a median value of 0.71 points. Within-patient variation calculated across the population using the other clinical outcome measures showed similar distributions, demonstrating two important findings: (1) quantifying clinical response based on a single follow-up visit may not accurately represent a patient’s response to a treatment and (2) the degree to which a patient’s disease fluctuates due to unknown factors varies from patient to patient.

Furthermore, when we compared outcomes determined at different follow-up visits (4, 12, 20, or 28 weeks after initial GOL treatment), we found that outcomes obtained from different follow-up visits and using different outcome measures had little or no correlation to one another (Fig. 2 b). Looking between different outcome metrics, for example, the change in lCRP after GOL treatment had a very low correlation with the changes in rSJC or rTJC after treatment (less than 0.3, regardless of which follow-up visits were considered). Even when comparing the same metric across different follow-up visits, the maximum correlation coefficient was only 0.76 (DAS_20WAG vs DAS_28WAG) (Additional file 1: Figure S1). The low correlations between outcomes calculated using a single follow-up visit further demonstrate the limitations of this approach. From these results, it is clear that the results of any genetic association study would be greatly impacted depending on which outcome measure was used (e.g., DAS or lCRP) and which clinical visits were used to calculate a patient’s response (e.g., 4WAG or 12WAG).

Because patients show clinically meaningful variations in disease state over time and because that variation results in low correlations between potential outcome measures, using a response measurement from a single follow-up visit may not sufficiently capture the efficacy of a treatment for a given patient. As a result, we explored the use of the repeated measurements for each individual over the course of the trial to identify a more representative response metric. We calculated the average response of each patient using multiple visits before (when available) and after initiation of GOL treatment. The relatively high correlations between the average response estimates (MNa) and the response outcomes obtained from single follow-up visits suggest that simply taking an average of the disease state over several clinical visits provides a more representative view of patients’ response to a given treatment (Fig. 2 b).

### Heritability estimates

**Fig. 3:**
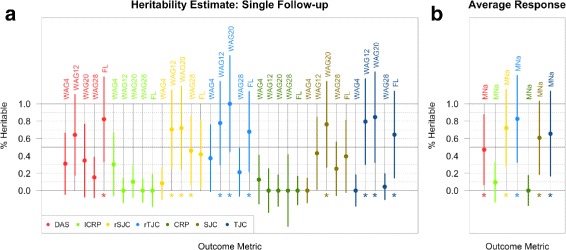
Heritability estimates of potential response outcomes. **a**, **b** GCTA heritability estimates of treatment response for transformed and raw clinical outcomes (**a**) using the change in disease state measured from a single follow-up visit and (**b**) using the mean difference in disease state after treatment. (* indicates *p*<0.05.) *CRP* C-reactive protein, *DAS* Disease Activity Score, *FL* difference between first and last measurements, *GOL* golimumab, *lCRP* log transform of C-reactive protein metric, *MNa* mean change in disease state before/after GOL, *rSJC* square root of SJC, *rTJC* square root of TJC, *SJC* swollen joint count, *TJC* tender joint count, *WAG* weeks after initial GOL treatment

Since genetic studies rely on a heritable clinical phenotype, we estimated the proportion of phenotypic variance explained by common single-nucleotide variations for both singular and averaged response statistics. The heritability estimates varied, both across phenotypes and between time points. At follow-up measurements 4, 12, 20, or 28 weeks after initial GOL treatment, the heritability estimate for the change in DAS was not significantly different from 0; however, when we used the difference between the final and initial measurements (weeks 100 and 0), the resulting heritability estimate was greater than 0. Similarly, for transformed SJC and TJC, the heritability estimate was significantly greater than 0 for only three of five time points used, for each measure (Fig. 3 a; Additional file 1: Table S1). After averaging phenotypic measurements over time, we found that MNa for DAS, rSJC, and rTJC all had heritability estimates significantly greater than 0 (Fig. 3 b). The transformed CRP estimate was non-significant for all five single measurement estimates, nor for the averaged response. These results suggest that simply averaging multiple measurements may be sufficient to reduce the noise and get a more reliable measure of drug response. Furthermore, we found that the heritability estimates for the transformed data were greater than for the raw data. We also estimated the heritability of these phenotypes while including the first four principal components of the genetic data to control for population stratification (Additional file 1: Figure S2). The results were very similar, with estimates showing a correlation of 0.93 with the original model for single time point outcomes (Additional file 1: Figure S3).

### Placebo response

**Fig. 4:**
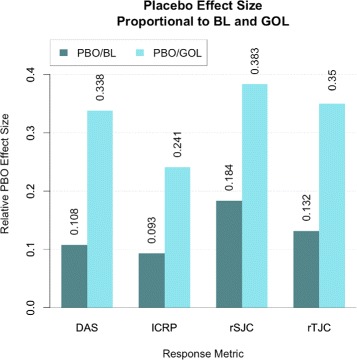
Relative magnitude of placebo effect. Population-level estimates of placebo effect for different clinical metrics. Percentage improvement of disease state from PBO treatment relative to BL (*dark bars*) and magnitude of PBO effect relative to magnitude of GOL effect (*light bars*). *BL* baseline disease state, *DAS* Disease Activity Score, *GOL* golimumab, *lCRP* log transform of C-reactive protein metric, *PBO* placebo, *rSJC* square root of SJC, *rTJC* square root of TJC, *SJC* swollen joint count, *TJC* tender joint count

Using the fixed estimates of baseline disease state (intercept), GOL effect size, and PBO effect size from the linear mixed model, we identified a statistically significant reduction in all disease metrics in patients who maintained their MTX regimen while enrolled in the PBO arm (Table 2). For DAS, the average pre-treatment disease state for the population was estimated to be 5.93, with GOL and PBO resulting in an average improvement of 1.89 and 0.64, respectively. The magnitude of the PBO effect resulted in, on average, a 10.8% improvement in DAS, which accounts for roughly 33.8% of the improvement in disease state that would otherwise be attributed to GOL treatment. Similarly, PBO resulted in a 9.3% improvement from baseline values for lCRP, and a 18.4% and 13.2% improvement from baseline values for rSJC and rTJC, respectively (Fig. 4). Importantly, the magnitude of the PBO effect relative to the GOL effect was larger for rSJC and rTJC than for lCRP (Fig. 4; rSJC = 38.3%; rTJC = 35%, lCRP = 24.1%).

**Table 2:** Golimumab and placebo effect estimates

Outcome	Fixed effect	Value	Standard error	*p* value
DAS	Baseline	5.931	0.057	0.000
	GOL	−1.892	0.054	1.760×10^−241^
	PBO	−0.640	0.058	5.277×10^−28^
lCRP	Baseline	1.246	0.022	0.000
	GOL	−0.482	0.024	7.423×10^−88^
	PBO	−0.116	0.024	1.479×10^−6^
rSJC	Baseline	3.752	0.063	0.000
	GOL	−1.799	0.059	3.150×10^−191^
	PBO	−0.690	0.065	9.436×10^−26^
rTJC	Baseline	4.992	0.077	0.000
	GOL	−1.876	0.068	1.149×10^−159^
	PBO	−0.657	0.074	6.040×10^−19^

Fixed effect estimates of baseline disease state, GOL effect size, and PBO effect size derived from linear mixed models that used various outcomes (e.g., DAS, lCRP, etc.) as response metric *DAS* Disease Activity Score, *GOL* golimumab, *lCRP* log transform of C-reactive protein metric, *PBO* placebo, *rSJC* square root of SJC, *rTJC* square root of TJC, *SJC* swollen joint count, *TJC* tender joint count

These results indicate that the active PBO influenced outcomes based on rSJC and rTJC more than the responses based on lCRP levels. Since DAS is a composite score that includes lCRP, rSJC, and rTJC, the PBO effect size for DAS fell between the estimates for the joint counts and inflammation marker. The change in lCRP levels after GOL treatment was less influenced by PBO than other potential outcomes, but it also had the lowest heritability estimate. Conversely, the change in rSJC and rTJC had significant heritability estimates, but was influenced by PBO to a greater extent. The change in DAS after GOL treatment was less influenced by PBO than rSJC or rTJC, but still had an appreciable heritable component. These results highlight the complexities of performing a study of this nature, which requires a heritable phenotype that reliably represents the characteristic of interest (i.e., response to GOL).

## Discussion

In the new era of precision medicine, pharmacogenetic studies may provide key insights that can be used to guide clinical decision-making. There are successful cases in oncology in which genotype-based companion diagnostics are used to stratify patients to receive the treatment likely to be most efficacious, resulting in greater overall survival and more cost-effective treatment regimens [27, 28]. However, this potential has not been realized for immunological diseases because such predictive genotypes have not been identified. In RA, for example, several studies have described genetic associations with response to expensive anti-TNF *α* biologics, but the results of these studies have not been reproducible [15–19]. While the limited sample sizes may contribute to this discrepancy, one often overlooked issue in these studies is the phenotype itself. The success of pharmacogenetic studies depends on many factors, not the least of which is the analysis of a phenotype with a significant heritable component. We sought to gain insight into the value of various clinical measurements in understanding individualized treatment response.

Our findings resulted in several useful insights that could guide the design and interpretation of future pharmacogenetic studies. First, we found that continuous response phenotypes often require a transformation to conform to the assumptions of many parametric statistical analysis approaches. Violations of these assumptions may produce unreliable *p* values and confound results from the study. Conversely, the use of non-parametric methods often leads to a loss in power, which would have to be compensated for by increasing the size (and cost) of the study. Second, measuring a patient’s disease state at a single time point after treatment is an insufficient and possibly misleading means of assessing their true response to that therapy. Many metrics, including those often used in RA, fluctuate over the course of a day or week and can be influenced by environmental factors. Collecting multiple measurements over time and controlling for non-genetic factors will better allow researchers to separate the signal from the noise. Third, the degree to which a response phenotype is heritable can vary depending on which metric is used. Using a response measurement that is not heritable would plague any genetic study from the outset. For RA, the changes in swollen or tender joints resulting from anti-TNF *α* treatment are estimated to be the most heritable response metrics by our analysis as well as a previous study [22]. Finally, we found that the magnitude of the PBO effect varied among different response metrics. Because some clinical measurements (e.g., pain used to assess TJC [29]) are subjective while others (e.g., inflammation markers) are not, they can be influenced to a greater or lesser extent by the idea that one is receiving treatment. Pain perception, for example, is known to be influenced by a range of factors from mood to attention [30–32].

Taken together, these results paint a complicated picture for the pharmacogenetics of anti-TNF *α* treatment. In our study, the change in the number of swollen or tender joints after treatment had the highest estimates of heritability, but were also the most influenced by PBO, improving the disease state by roughly 15% and accounting for 35–40% of the improvement seen during anti-TNF *α* treatment. While all patients had previously been on a MTX regimen, it is possible that a portion of this PBO effect results from more attentive care and greater adherence to DMARD treatment upon entering the clinical trial. Conversely, the phenotype least influenced by active PBO treatment, change in CRP, was also the least heritable. As a composite score, DAS represents an adequate balance between subjective and non-subjective measurements and is useful in pharmacogenetic studies as a heritable metric that is more robust to PBO response. Our results also show a (statistical) benefit from more frequent monitoring of patients, though this could prove implausible if it involves expensive molecular and radiographic tests.

While we gained many valuable insights from this study, there were still limitations to our retrospective analysis. Our sample size remains small for genome-wide association and heritability studies since the availability of large, deeply phenotyped clinical cohorts is very limited. However, by reducing noise and accounting for known sources of variation in our study, we were able to improve our ability to detect meaningful signals in the data. Additionally, as with most retrospective analyses of clinical trials, we were limited by the trial design. Roughly two-thirds of patients were randomized to GOL from the outset of the trial, meaning that these patients were never treated with PBO alone in the trial and we cannot directly estimate their individual PBO response. Furthermore, we cannot say unequivocally how well their disease was maintained prior to enrollment in the trial. Thus, some of the PBO response could be a result of more attentive care and greater adherence to the DMARD regimen.

Future trials in which all patients are treated with PBO and the baseline disease state is monitored and maintained could ameliorate some of these issues. Another consideration in our findings is that both RA patients and drug developers could benefit from frequent monitoring of joint conditions, which could be facilitated by smartphone-enabled health apps. It is possible that more frequent self-reports of disease state (e.g., from health-monitoring apps and/or devices) could yield a sufficiently high-resolution dataset to provide a better picture of a patient’s disease state and lead to useful clinical insights. Such study designs would require specialized longitudinal analysis approaches to account for the serial correlation between measurements on a single individual. Future studies could also consider objective molecular proxies for disease activity; however, recurrent molecular testing could prove costly and would limit the frequency of follow-ups in clinical studies. Because some types of data collected may not conform to the assumptions of basic parametric statistical methods, even after transformation, the use of permutations, bootstrapping, Monte Carlo simulations, and other sophisticated statistical methods may be necessary for accurately interpreting the data.

## Conclusions

While concerted efforts have been made to assemble larger and larger genetic studies, attention to the clinical phenotype is often lacking, complicating the effort to identify biomarkers that can guide treatment decisions by physicians. Our study suggests that a comprehensive evaluation of clinical response phenotypes can result in more effective and efficient pharmacogenetic studies. Using the correct statistical methods, accounting for within-patient variation, and using a heritable phenotype that is not confounded by PBO response will result in consistent results that truly advance precision medicine.

## Additional file

Additional file 1 Supplemental figures and descriptions for each figure, and supplemental table 1 and a description of the data. (PDF 5149 kb)

## Abbreviations

ACPA: Anti-citrulinated peptide antibody
ACR: American College of Rheumatology
BMI: Body mass index
CRP: C-reactive protein
DAS: Disease Activity Score
DMARD: Disease modifying anti-rheumatic drug
EE: Early escape
ESR: Erythrocyte sedimentation rate
FL: Difference between first and last measurements
GCTA: Genome-wide complex trait analysis toolkit
GOL: Golimumab
GRM: Genetic relationship matrix
lCRP: Log transform of CRP metric
MNa: Mean treatment response (calculated from area under response vs time curve)
MTX: Methotrexate
NE: Non-early escape
PBO: Placebo
PBO-EE: Placebo plus early escape protocol
PBO-NE: Placebo protocol (no early escape)
RA: Rheumatoid arthritis
RF: Rheumatoid factor
RMSE: root-mean-square error
rSJC: Square root of SJC
rTJC: Square root of TJC
SD: Standard deviation
SJC: Swollen joint count
TJC: Tender joint count
VASGH: Visual analog scale for global health
WAG: Weeks after golimumab initiation
