# Supplementary material for: Comprehensive analysis of treatment response phenotypes in rheumatoid arthritis for pharmacogenetic studies
Source: Arthritis Res Ther. 2017 May 12;19:90. doi: 10.1186/s13075-017-1299-8 (PMC5427602; doi:10.1186/s13075-017-1299-8)
Supplement: Additional file 1 — Supplemental figures and descriptions for each figure, and supplemental table 1 and a description of the data. (PDF 5149 kb) [file 13075_2017_1299_MOESM1_ESM.pdf]

Comprehensive Analysis of Treatment Response Phenotypes in Rheumatoid Arthritis for Pharmacogenetic Studies

Authors:

Standish K[1,2,3], Huang CC[4], Curran M[4], Schork NJ[2,3]

[1]Biomedical Sciences Graduate Program

[2]University of California, San Diego

[3]J. Craig Venter Institute

[4]Janssen R&D, LLC

Table 1

	G	P	PE
Num. Patients	287	99	50
Female* (%)	231 (0.8)	72 (0.73)	41 (0.82)
Age* (SD)	51.92 (11.94)	52.77 (11.17)	49.24 (11.84)
Disease Duration* (SD)	7.19 (6.82)	7.64 (8.16)	6.56 (6.06)
BMI* (SD)	27.19 (5.74)	26.88 (5.36)	26.73 (6.65)
Initial DAS* (SD)	5.97 (0.81)	5.87 (1.02)	5.93 (0.8)
RF Positive* (%)	265 (0.92)	91 (0.92)	45 (0.9)
ACPA Positive* (%)	262 (0.91)	92 (0.93)	48 (0.96)
Num. Visits* (SD)	240 (0.84)	85 (0.86)	43 (0.86)
Num. Removed^ (%)	15.03 (2.7)	14.65 (3.18)	15.76 (0.72)

Summary of Clinical Trial Patients and Arms.

GOL includes patients randomized to GOL at week 0. PBO-NE includes patients randomized to PBO at week 0 and did not qualify for "early escape" protocol. PBO-EE includes patients randomized to PBO at week 0, but did qualify for "early escape" protocol.

^Patients from any arm who dropped out within 4 weeks of initial GOL treatment were removed from analyses.

Abbreviations: GOL=Golimumab, PBO=Placebo, NE=Non-Early Escape, EE=Early Escape, BMI=Body-Mass Index, DAS=Disease Activity Score, RF=Rheumatoid Factor, ACPA=Anti-Citrulinated Peptide Antibody, SD=Standard Deviation

Figure 1

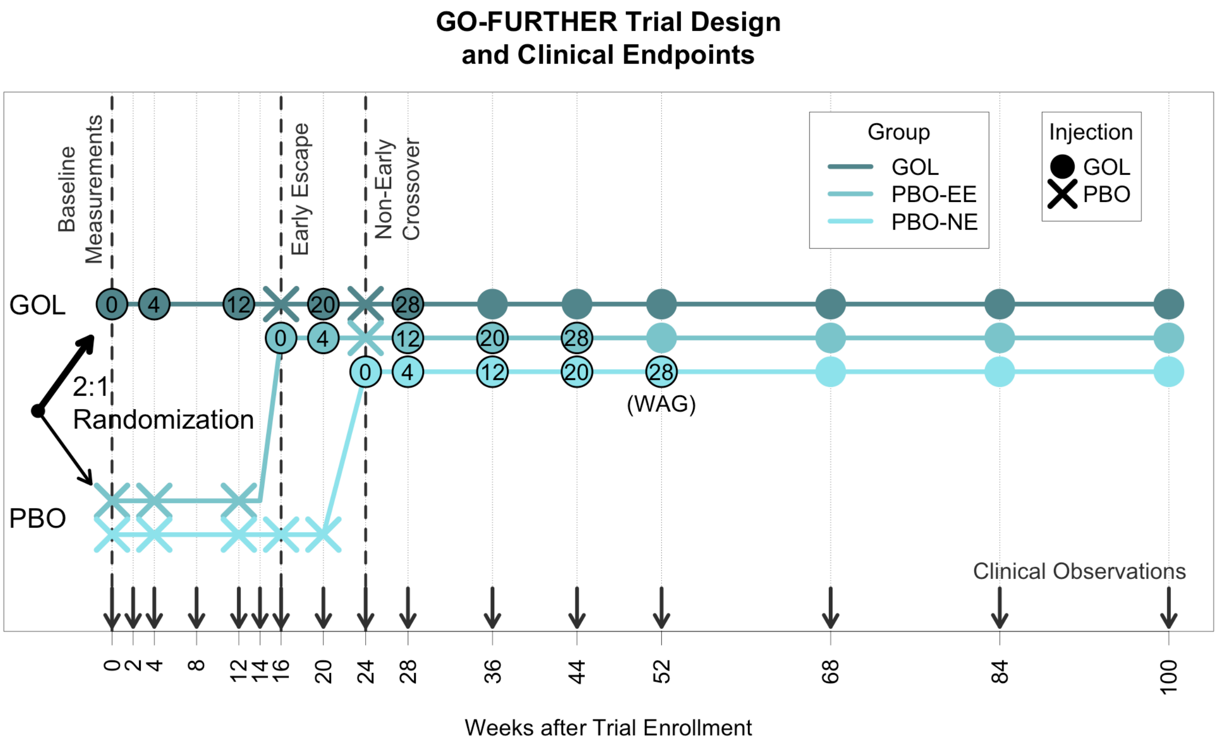

GO-FURTHER Trial Design and Clinical Endpoints

Longitudinal design for GO-FURTHER Phase III clinical trial. Patients were initially randomized (2:1) to GOL or PBO arms of the trial. GOL patients were initiated onto golimumab treatment at week 0. PBO patients were treated with placebo at week 0 and were treated with golimumab at week 24 (PBO-NE) or week 16 (PBO-EE). Injections and clinical observations occurred as indicated.

Abbreviations: GOL=Golimumab, PBO=Placebo, NE=Non-Early Escape, EE=Early Escape, WAG=Weeks After Initial GOL Treatment

Figure 2

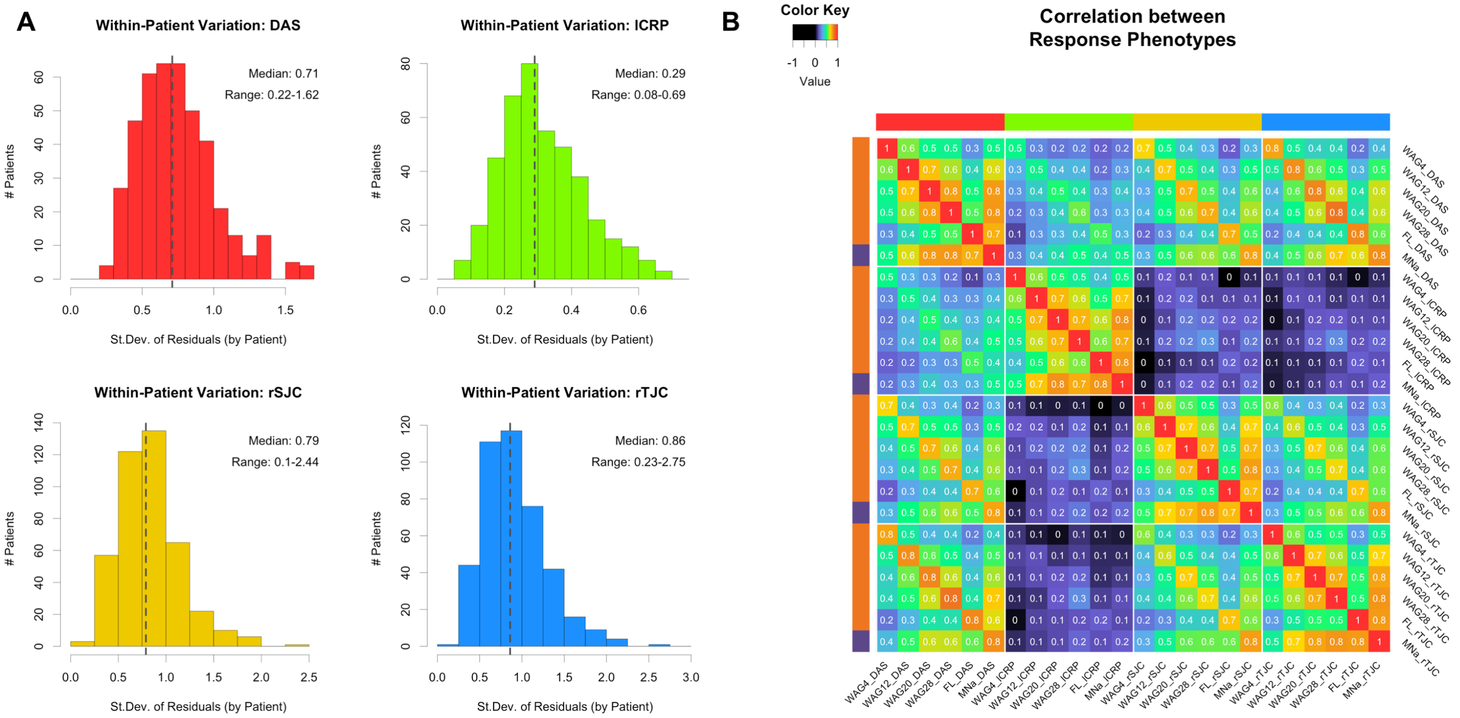

Variability in Potential Response Outcomes

A. Distribution across cohort of within-patient RMSE of regression model for transformed phenotypes.

B. Pairwise Pearson correlation between potential clinical outcomes using various response metrics and follow-up visits.

Abbreviations: WAG=Weeks After Initial GOL Treatment, FL=Difference of First and Last Measurements, DAS=Disease Activity Score, lCRP=Logarithmic Transform of CRP, rSJC/rTJC=Square Root Transform of SJC/TJC

Figure 3

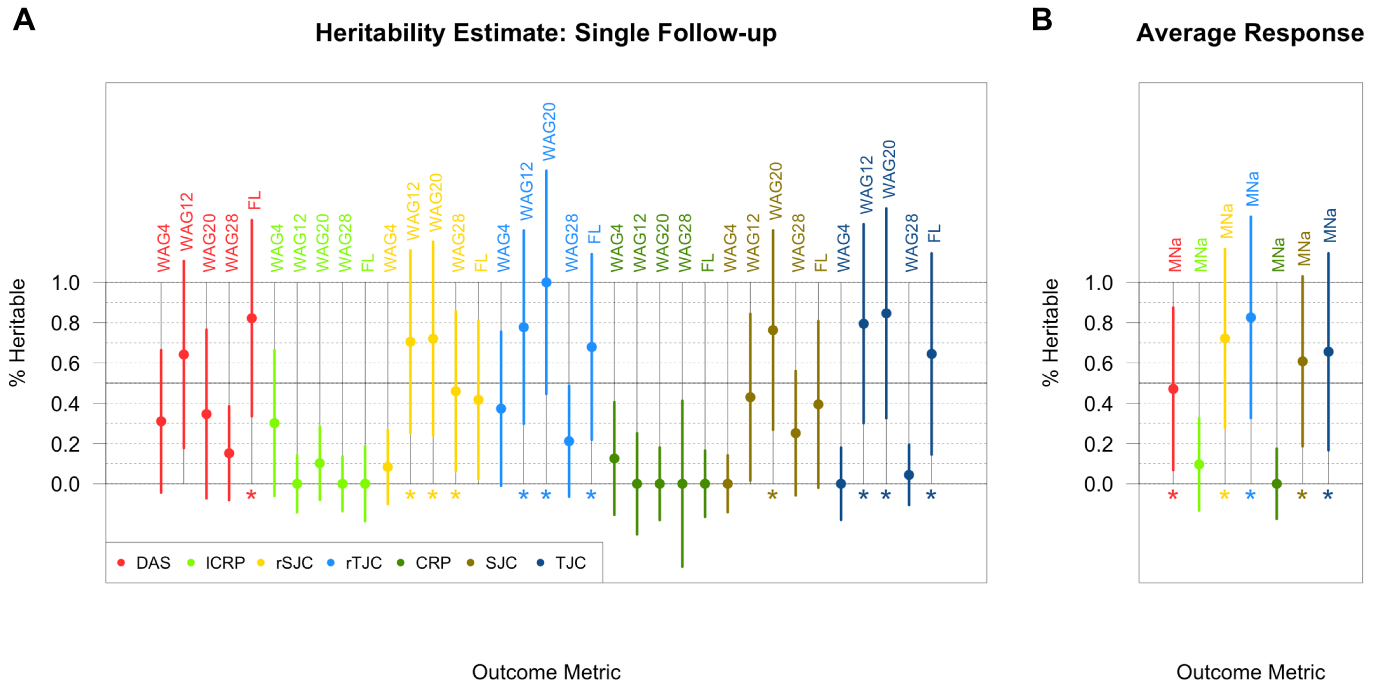

Heritability Estimates of Potential Response Outcomes

A,B) GCTA Heritability Estimates using difference between

A) single measurements and

B) using mean difference in disease state after treatment.

(* indicates $p<0.05$)

Abbreviations: WAG=Weeks After Initial GOL Treatment, FL=Difference of First and Last Measurements, DAS=Disease Activity Score, CRP=C-Reactive Protein, SJC=Swollen Joint Count, TJC=Tender Joint Count, lCRP=Logarithmic Transform of CRP, rSJC/rTJC=Square Root Transform of SJC/TJC, MNa=Mean Change in Disease State before/after GOL

Table 2

Outcome	Fixed Effect	Estimate	Std.Error.	P
**DAS**	Baseline	5.931407905	0.056829796	0
	GOL	-1.892185435	0.054444894	1.76E-241
	PBO	-0.640005854	0.058044914	5.28E-28
**lCRP**	Baseline	1.246416685	0.022059025	0
	GOL	-0.481715207	0.023844275	7.42E-88
	PBO	-0.116076691	0.024088025	1.48E-06
**rSJC**	Baseline	3.751625884	0.062519915	0
	GOL	-1.798831296	0.058794177	3.15E-191
	PBO	-0.68951738	0.065418022	9.44E-26
**rTJC**	Baseline	4.991614691	0.077000367	0
	GOL	-1.876113607	0.06759471	1.15E-159
	PBO	-0.657052286	0.073653083	6.04E-19

Golimumab and Placebo Effect Estimates

Placebo Effect Size Estimates. Fixed effect estimates of baseline disease state, GOL effect size, and PBO effect size derived from linear mixed models that used various outcomes (e.g., DAS, lCRP, etc.) as response metric.

Abbreviations: GOL=Golimumab, PBO=Placebo, DAS=Disease Activity Score, lCRP=Logarithmic Transform of CRP, rSJC/rTJC=Square Root Transform of SJC/TJC, P=p-value

Figure 4

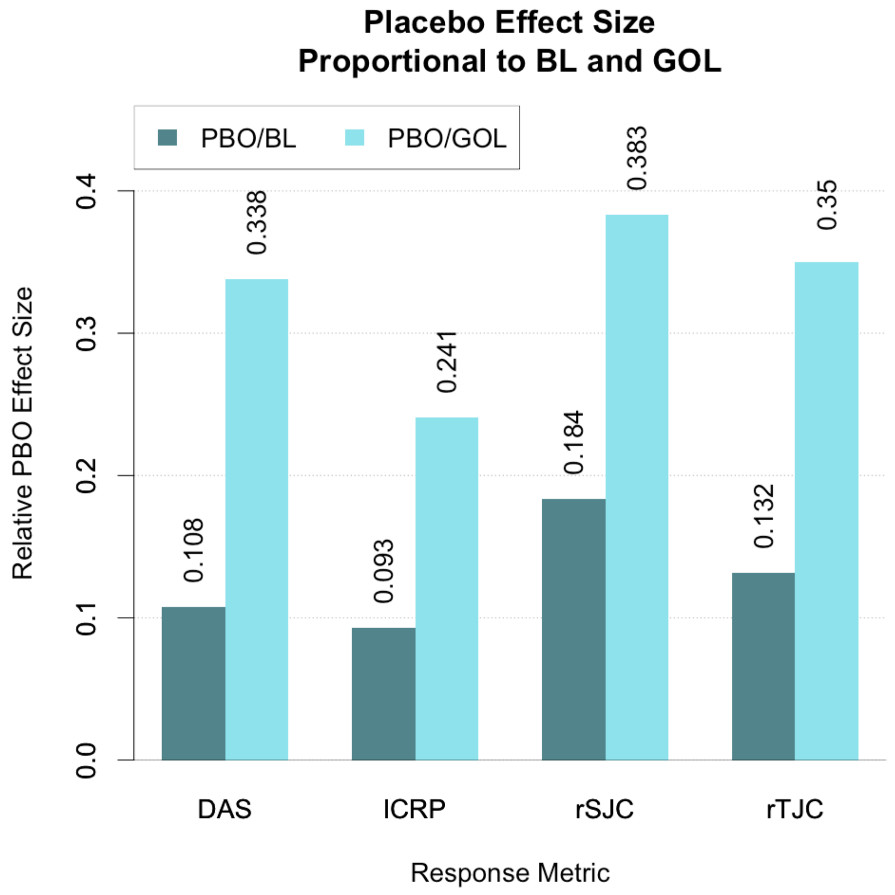

<u>**Relative Magnitude of Placebo Effect**</u>

Percent improvement of phenotypes from PBO treatment relative to BL (dark bars) and magnitude of PBO effect relative to magnitude of GOL effect (light bars).

Abbreviations: BL=Baseline Disease State, GOL=Golimumab, PBO=Placebo, DAS=Disease Activity Score, lCRP=Logarithmic Transform of CRP, rSJC/rTJC=Square Root Transform of SJC/TJC

Supplemental Material

Comprehensive Analysis of Treatment Response Phenotypes in Rheumatoid Arthritis for Pharmacogenetic Studies

Authors:

Standish K[1,2,3], Huang CC[4], Curran M[4], Schork NJ[2,3]

[1]Biomedical Sciences Graduate Program

[2]University of California, San Diego

[3]J. Craig Venter Institute

[4]Janssen R&D, LLC

Supp. Figure 1

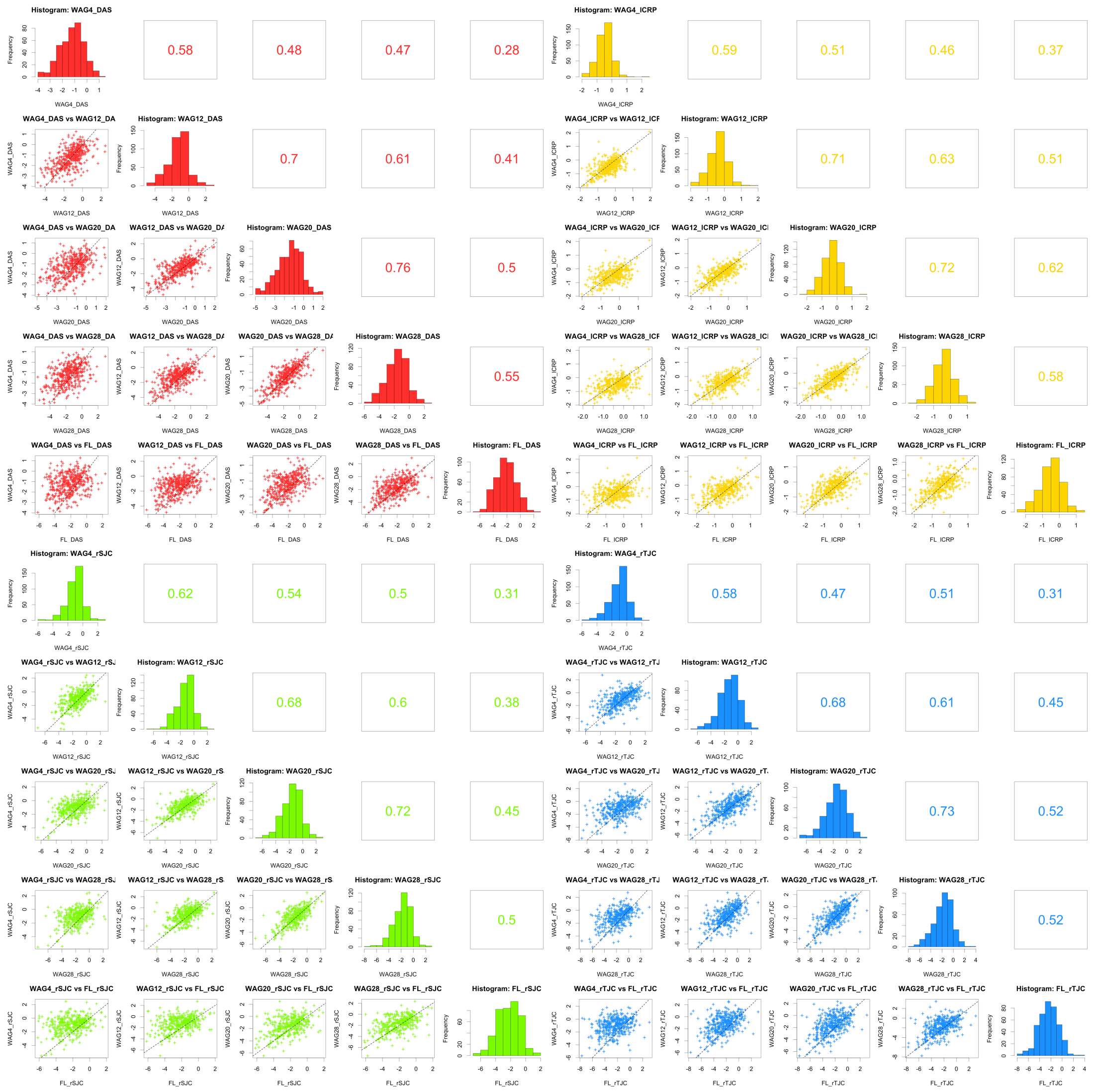

Pairwise correlation of Single Measurements

A. Pairwise correlation of change in DAS using measurements at single time points. (red)

B. Pairwise correlation of change in lCRP. (yellow)

C. Pairwise correlation of change in rSJC. (green)

D. Pairwise correlation of change in rTJC. (blue)

Abbreviations: WAG=Weeks After Initial GOL Treatment, FL=Difference of First and Last Measurements, DAS=Disease Activity Score, CRP=C-Reactive Protein, SJC=Swollen Joint Count, TJC=Tender Joint Count, lCRP=Logarithmic Transform of CRP, rSJC/rTJC=Square Root Transform of SJC/TJC

Supp. Figure 2

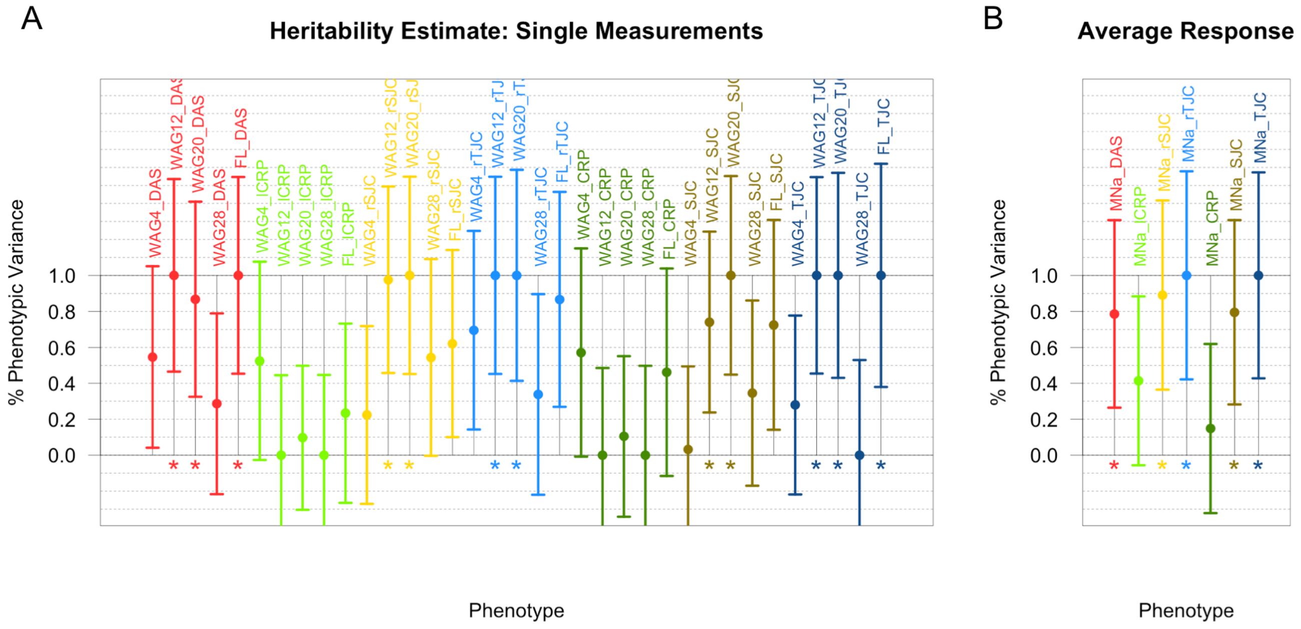

<u>Heritability Estimates (PC4 model)</u>

A,B) GCTA Heritability Estimates using difference between

A) single measurements and B) using mean difference in disease state after treatment. (* indicates $p<0.05$)

Abbreviations: WAG=Weeks After Initial GOL Treatment, FL=Difference of First and Last Measurements, DAS=Disease Activity Score, CRP=C-Reactive Protein, SJC=Swollen Joint Count, TJC=Tender Joint Count, lCRP=Logarithmic Transform of CRP, rSJC/rTJC=Square Root Transform of SJC/TJC, MNa=Mean Change in Disease State before/after GOL

Supp. Figure 3

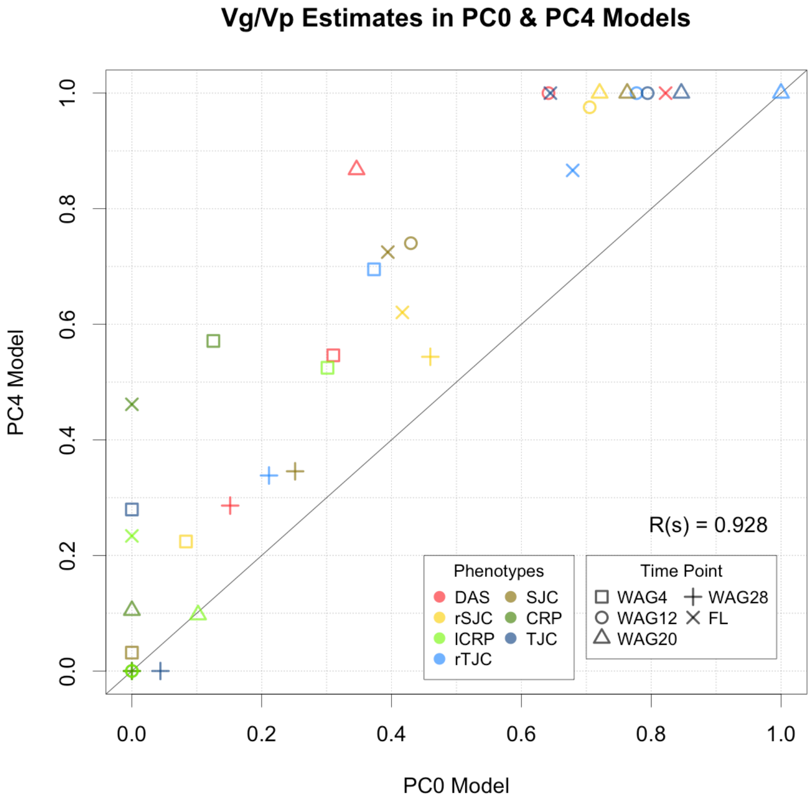

PC0 vs PC4 model

Heritability estimates for PC0 vs PC4 models for single time point phenotypes

Abbreviations: WAG=Weeks After Initial GOL Treatment, FL=Difference of First and Last Measurements, DAS=Disease Activity Score, CRP=C-Reactive Protein, SJC=Swollen Joint Count, TJC=Tender Joint Count, lCRP=Logarithmic Transform of CRP, rSJC/rTJC=Square Root Transform of SJC/TJC, MNa=Mean Change in Disease State before/after GOL, MNcd=Cohen's D-Statistic, PRC=Percent Change, Bwk=Disease Trajectory after GOL, VARwk=Post-GOL Variance

Supp. Table 1

Phenotype	Time	VgVp	SE	P-value
DAS	WAG4	0.546	0.505	9.78E-02
	WAG12	1.000	0.535	3.63E-01
	WAG20	0.868	0.542	5.89E-02
	WAG28	0.286	0.503	4.94E-01
	FL	1.000	0.547	8.98E-03
	MNa	0.786	0.522	2.69E-02
lCRP	WAG4	0.525	0.551	4.57E-01
	WAG12	0.000	0.445	5.69E-02
	WAG20	0.097	0.401	4.01E-01
	WAG28	0.000	0.447	3.19E-02
	FL	0.234	0.499	2.99E-01
	MNa	0.414	0.470	1.29E-01
rSJC	WAG4	0.224	0.494	7.98E-03
	WAG12	0.976	0.518	4.69E-01
	WAG20	1.000	0.548	2.28E-01
	WAG28	0.544	0.547	3.12E-01
	FL	0.621	0.520	2.99E-03
	MNa	0.891	0.526	9.98E-03
rTJC	WAG4	0.695	0.552	2.12E-01
	WAG12	1.000	0.548	6.99E-03
	WAG20	1.000	0.586	2.99E-03
	WAG28	0.338	0.558	2.43E-01
	FL	0.866	0.598	4.34E-01
	MNa	1.000	0.578	1.20E-02
CRP	WAG4	0.571	0.579	1.52E-01
	WAG12	0.000	0.485	7.98E-03
	WAG20	0.105	0.447	4.49E-02
	WAG28	0.000	0.498	3.99E-02
	FL	0.462	0.577	4.63E-01
	MNa	0.149	0.470	3.37E-01
SJC	WAG4	0.032	0.462	1.19E-01
	WAG12	0.740	0.503	1.96E-01
	WAG20	1.000	0.552	6.99E-03
	WAG28	0.346	0.515	2.18E-01
	FL	0.725	0.583	9.38E-02
	MNa	0.795	0.513	2.99E-02
TJC	WAG4	0.280	0.498	2.54E-01
	WAG12	1.000	0.546	4.99E-03
	WAG20	1.000	0.570	5.99E-03
	WAG28	0.000	0.530	4.63E-01
	FL	1.000	0.620	2.20E-02
	MNa	1.000	0.572	1.30E-02

Heritability Estimates.

GCTA Heritability Estimates using difference between single measurements and mean difference in disease state after treatment.

Abbreviations: VgVp=Proportion of Phenotypic Variance explained by SNPs, SE=Standard Error of VgVp Estimate, P-value=p-value based on permutations, WAG=Weeks After Initial GOL Treatment, FL=Difference of First and Last Measurements, MNa=Mean Change in Disease State before/after GOL, DAS=Disease Activity Score, CRP=C-Reactive Protein, SJC=Swollen Joint Count, TJC=Tender Joint Count, lCRP=Logarithmic Transform of CRP, rSJC/rTJC=Square Root Transform of SJC/TJC
